# Microbial Population Dynamics and Ecosystem Functions of Anoxic/Aerobic Granular Sludge in Sequencing Batch Reactors Operated at Different Organic Loading Rates

**DOI:** 10.3389/fmicb.2017.00770

**Published:** 2017-05-01

**Authors:** Enikö Szabó, Raquel Liébana, Malte Hermansson, Oskar Modin, Frank Persson, Britt-Marie Wilén

**Affiliations:** ^1^Division of Water Environment Technology, Department of Civil and Environmental Engineering, Chalmers University of TechnologyGothenburg, Sweden; ^2^Department of Chemistry and Molecular Biology, University of GothenburgGothenburg, Sweden

**Keywords:** microbial community dynamics, aerobic granular sludge, microbial functional groups, ecosystem functions, organic loading rate, nitrogen removal, wastewater treatment, sequencing batch reactors

## Abstract

The granular sludge process is an effective, low-footprint alternative to conventional activated sludge wastewater treatment. The architecture of the microbial granules allows the co-existence of different functional groups, e.g., nitrifying and denitrifying communities, which permits compact reactor design. However, little is known about the factors influencing community assembly in granular sludge, such as the effects of reactor operation strategies and influent wastewater composition. Here, we analyze the development of the microbiomes in parallel laboratory-scale anoxic/aerobic granular sludge reactors operated at low (0.9 kg m^-3^d^-1^), moderate (1.9 kg m^-3^d^-1^) and high (3.7 kg m^-3^d^-1^) organic loading rates (OLRs) and the same ammonium loading rate (0.2 kg NH_4_-N m^-3^d^-1^) for 84 days. Complete removal of organic carbon and ammonium was achieved in all three reactors after start-up, while the nitrogen removal (denitrification) efficiency increased with the OLR: 0% at low, 38% at moderate, and 66% at high loading rate. The bacterial communities at different loading rates diverged rapidly after start-up and showed less than 50% similarity after 6 days, and below 40% similarity after 84 days. The three reactor microbiomes were dominated by different genera (mainly *Meganema, Thauera, Paracoccus*, and *Zoogloea*), but these genera have similar ecosystem functions of EPS production, denitrification and polyhydroxyalkanoate (PHA) storage. Many less abundant but persistent taxa were also detected within these functional groups. The bacterial communities were functionally redundant irrespective of the loading rate applied. At steady-state reactor operation, the identity of the core community members was rather stable, but their relative abundances changed considerably over time. Furthermore, nitrifying bacteria were low in relative abundance and diversity in all reactors, despite their large contribution to nitrogen turnover. The results suggest that the OLR has considerable impact on the composition of the granular sludge communities, but also that the granule communities can be dynamic even at steady-state reactor operation due to high functional redundancy of several key guilds. Knowledge about microbial diversity with specific functional guilds under different operating conditions can be important for engineers to predict the stability of reactor functions during the start-up and continued reactor operation.

## Introduction

The aerobic granular sludge process is an emerging technology for the treatment of domestic as well as industrial wastewater streams (Khan et al., 2013). Granular sludge can be considered as a special case of biofilm, where self-immobilized bacterial cells form strong, dense and well-settling aggregates (Show et al., 2012). The properties of the granules allow the process to handle high loading rates and toxic substances in a compact bioreactor. Urbanization results in increasing pollutant loads and often limited availability to expand existing treatment plants, which makes this effective, low-footprint process an attractive alternative (Khan et al., 2013). The dense structure of the granular sludge also creates substrate gradients in the granule, which in turn results in different niches for different functional groups (Winkler et al., 2013). Studies have shown that aerobic and anaerobic metabolic activities can co-exist in granules, like the simultaneous removal of organic compounds, nitrogen and phosphorus (de Kreuk et al., 2005; Li et al., 2014), simultaneous nitrification and denitrification (Third et al., 2003; Yang et al., 2003), or simultaneous nitritation and anammox (Nielsen et al., 2005; Vlaeminck et al., 2010).

Although the aerobic granular sludge process was first reported in the early 1990s (Mishima and Nakamura, 1991; Morgenroth et al., 1997), we still have an incomplete understanding of the parameters that drive the development of the microbial community composition of aerobic granules (Weissbrodt et al., 2012; Bindhu and Madhu, 2014). Most studies investigate the effects of operational parameters by analyzing the community composition in samples obtained at a single sampling date during steady-state operation (e.g., Ebrahimi et al., 2010; Gonzalez-Gil and Holliger, 2011; Cydzik-Kwiatkowska, 2015). Only a few reports assess the temporal variation of the microbiome (Li et al., 2008; Weissbrodt et al., 2013; Lv et al., 2014). However, steady-state process performance does not necessitate stable microbial community composition: long-term studies of full-scale activated sludge treatment plants revealed strong variation in the bacterial population and a gradual succession away from initial conditions (Wells et al., 2011; Fredriksson, 2013; Kim et al., 2013). The temporal variations of dominant and rare taxa were reported to show different patterns, the dominant taxa being more persistent (Kim et al., 2013). Nonetheless, dominant taxa were strongly affected by the availability of organic carbon in the influent.

The effect of volumetric organic loading rate (OLR) on granulation has been extensively studied, but only few experiments have been conducted using low-strength influent similar to domestic wastewater (OLR < 2.0 kg COD m^-3^d^-1^, COD_in_ < 600–800 mg L^-1^) (Tchobanoglous et al., 2004; Henze et al., 2008; Somlyódy and Patziger, 2012; Wagner and da Costa, 2013). The number of studies on population dynamics and community assembly at different OLRs is even lower. Li et al. (2008) studied aerobic granular sludge reactors operated for COD removal at loading rates of 1.5, 3.0, and 4.5 kg m^-3^d^-1^, and found that at different OLRs the bacterial populations changed at different rates. The highest loading rate resulted in the lowest diversity, but the same dominant genera (*Zoogloea, Thauera, Pseudomonas, Flavobacterium*, and a *Comamonadaceae* related genus) were found in all three reactors, although in different proportions.

Our aim was to study the impact of OLR on the development of the microbiomes in reactors operated for COD and nitrogen removal. Low (0.9 kg m^-3^d^-1^), moderate (1.9 kg m^-3^d^-1^), and high (3.7 kg m^-3^d^-1^) OLRs were tested. Three laboratory-scale sequencing batch reactors (SBRs) were run in parallel, the process performance was monitored for 12 weeks, and the bacterial population was analyzed by high-throughput amplicon sequencing (Illumina MiSeq) that allowed the investigation of the temporal variation and the functional redundancy at high resolution. Microbial diversity and functional redundancy have implications for the operational stability and performance of biological reactor. From an engineering perspective, it is therefore important to study how the microbiome develops in reactors operated under different conditions.

## Materials and Methods

### Setup and Operation of Anoxic/Aerobic SBRs

Three laboratory-SBRs with a working volume of 3 l were operated for 12 weeks, aiming for the removal of organic carbon and nitrogen (without enhanced biological phosphorus removal). Each cycle was 4 h long, and consisted of 5 min filling, 55 min anoxic phase, 143–171 min aerobic phase, 2–30 min settling, 5 min withdrawal and 2 min idle phase. The initial anoxic phase was applied to enhance denitrification (Val del Río et al., 2013), especially during the start-up when the granule diameter was not large enough to enable simultaneous nitrification and denitrification. During the anoxic phase the reactors were not mixed. The settling time was gradually decreased (Supplementary Figure S1) to avoid extensive biomass wash-out, and the aerobic phase was adjusted to permit an even, 4 h cycle length. The air was introduced from the bottom of the reactor through a diffusor stone (pore size 1 μm) with a superficial upflow air velocity of 1.5 cm s^-1^, which has resulted in saturated dissolved oxygen concentrations already after a few minutes in the aerobic phase. The reactors were seeded with aerobic/anoxic activated sludge from a full-scale treatment plant (Gryaab, Gothenburg, Sweden), where phosphorus is removed chemically and thus the sludge lacks phosphorus accumulating organisms. The influent was introduced at the bottom of the reactor, the effluent was withdrawn 63 cm from the bottom, resulting in a volume exchange ratio of 43% and a HRT of 9.3 h.

### Composition of the Influent Wastewater

The influent consisted of a 50–50% mixture of synthetic and diluted real wastewater. A simplified scheme of the reactor design is shown in Supplementary Figure S7. The diluted real wastewater (six times diluted sludge liquor from dewatering centrifuges after anaerobic digestion) served as the source of nitrogen, the ammonium concentration after dilution was 174 ± 12 mg L^-1^. Diluted sludge liquor was used because it has stable composition with very low COD concentration (<10 mg L^-1^), hence contributing insignificantly to the OLR, and contains suspended particles, hence mimicking real domestic wastewater. The nitrogen loading rate (NLR) was 0.2 kg NH_4_-N m^-3^d^-1^ in all three reactors. The composition of the synthetic wastewater is given in **Table 1**, the parameters of the influent are given in **Table 2**. The OLR was 3.7, 1.9, and 0.9 kg COD m^-3^d^-1^ in R1, R2, and R3, respectively, resulting in COD:N:P ratios of 100:6:1, 100:12:1, and 100:24:1. The reactor pH was measured and logged on-line, but neither the pH nor the temperature was regulated. These parameters varied between pH 7.0 and 9.0, and between 19°C and 21°C, respectively. In this study, acetate was used as the COD source. It should be noted that real domestic wastewater has a more complex composition of organic matter contributing to the COD.

**Table 1:** Composition of the synthetic wastewater.

	R1	R2	R3
NaCH_3_COO (mmol L^-1^)	27.0	12.9	5.6
CH_3_COOH (mmol L^-1^)	15.2	7.3	3.1
K_2_HPO_4_ (mg L^-1^)	71.0	35.5	17.8
CaCl_2_ (mg L^-1^)	22.1	22.1	22.1
MgSO_4_ × 7H_2_O (mg L^-1^)	24.4	24.4	24.4
FeSO_4_ × 7H_2_O (mg L^-1^)	19.5	19.5	19.5
Micronutrients (mL L^-1^)^∗^	1	1	1

*^∗^The recipe of the micronutrient solution can be found in Tay et al. (2001).*

**Table 2:** Influent parameters (mixture of synthetic and real wastewater).

	R1	R2	R3
OLR (kg m^-3^d^-1^)	3.7	1.9	0.9
NLR (kg m^-3^d^-1^)	0.2	0.2	0.2
COD:N ratio	100:6	100:12	100:24
NH_4_-N (mg L^-1^)	85 ± 6	85 ± 6	85 ± 6
COD (mg L^-1^)	1416 ± 14	712 ± 14	346 ± 14

### Sample Collection and Chemical Analysis

The reactors were sampled three times per week: the height of the settled sludge bed was measured, biomass samples were taken for DNA analysis, and effluent samples were taken for suspended solids (SS), total organic carbon (TOC), total nitrogen (TN), ammonium (NH_4_-N), nitrite (NO_2_-N), and nitrate (NO_3_-N) analyses. The effluent SS were analyzed according to standard methods (APHA, 1995). TOC and TN were analyzed using a Shimadzu TOC analyzer. Effluent chemical oxygen demand (COD) concentration was calculated from the measured TOC values (2.66 mg COD = 1 mg TOC). Ammonium, nitrite and nitrate were analyzed using Dionex ICS-900 ion chromatographs.

### Carbon and Nitrogen Conversions within a SBR Cycle

To investigate how carbon and nitrogen was converted within the cycles of the three SBRs, cycle studies were performed on day 77. Twelve samples were taken during a cycle, six under the anoxic phase, and six under the aerobic phase. Liquid samples were withdrawn from the reactor with a flexible plastic tube (ø 1 cm) attached to a syringe, and then immediately centrifuged, filtered (0.45 μm) and stored at -20°C until analysis (TOC, TN, NH_4_-N, NO_2_-N, NO_3_-N). Nitrogen assimilation was estimated based on the biomass production rate assuming a biomass composition of C_5_H_7_O_2_N. Average granule diameter was determined by measuring 240 individual granules with a caliper (80 from each reactor).

### DNA Extraction, PCR Amplification, and High Throughput Amplicon Sequencing

DNA was extracted from the biomass samples using the FastDNA Spin Kit for Soil (MP Biomedicals) following the manufacturer’s protocol. PCR amplification of the 16S rRNA genes was carried out in duplicates in a Biometra T3000 Thermocycler, starting with 5 min enzyme activation at 95°C, followed by 30 cycles of denaturation (95°C, 20 s), annealing (50°C, 15 s), and elongation (68°C, 60 s), and finishing by a 10 min final elongation at 68°C. The reaction mixture consisted of 20 ng template, AccuPrime Pfx Supermix (Life Technologies), and 1 μM forward (515F) and 1 μM reverse (806R) primers, dual-indexed according to Kozich et al. (2013) The duplicate PCR products were pooled, the DNA concentration was normalized and the samples were purified using the Agencourt AMPure system (Beckman Coulter). The PCR products were multiplexed and diluted with Tris-Cl (pH 8.5, 0.1% Tween20) for a final concentration of 0.6 ng μL^-1^, as measured by Qubit 2.0 (Life Technologies). The expected concentration and size of the pooled PCR product was confirmed by TapeStation 2200 (Agilent Technologies). PhiX control library was spiked in at 7.5%. Sequencing was performed on an Illumina MiSeq using the MiSeq Reagent Kit v2. The sequence reads were processed and assigned to taxonomy as published in Albertsen et al. (2015) and were subsequently analyzed in R (R Core Team, 2016). Non-metric multi-dimensional scaling (NMDS) ordination and heatmaps were created using the package ampvis (Albertsen et al., 2015). The NMDS ordination was based on Bray-Curtis dissimilarity matrix calculated from the square root transformed, Wisconsin double standardized relative abundance of OTUs. Tukey boxplots were drawn with the package ggplot2 (Wickham, 2009). For assessment of diversity, Margalef richness and Pielou’s evenness indices were calculated based on the number and relative abundance of OTUs, using Microsoft Excel.

### Fluorescence *In Situ* Hybridization and Confocal Laser Scanning Microscopy (FISH-CLSM)

Intact granules were harvested from the reactors after 55 days of operation for FISH-CLSM analysis. Granules were fixed by immersion into 4% paraformaldehyde for 8 h at 4°C, followed by washing twice with PBS and stored in PBS/ethanol (50:50) at -20°C until use. For cryosectioning, granules were embedded in O.C.T. Compound (VWR, Radnor, PA, USA) and incubated overnight at 4°C in individual plastic containers. Thereafter, each granule was frozen solid in blocks in a dry ice fume chamber and stored at -80°C until use. Granules were cut into 10–20 μm thick cryosections with a HM550 microtome cryostat (MICROM International GmbH, Germany) at -20°C. The cryosections were collected on SuperFrost^®^ Plus Gold microscope slides (Menzel GmbH, Germany) and stored at -20°C. Before FISH, the cryosections on the slides were framed with a hydrophobic barrier using a Liquid Blocker Mini PAP Pen (Life Technologies) and the glass slides were covered with a thin layer of agarose (1%) to preserve the cryosections integrity. After dehydration in an ethanol series (50, 80, and 96% v/v), FISH was performed at 46°C for 2 h (Manz et al., 1992). Probes were 5′-labeled with Cy3. Probe specifications and hybridization conditions are shown in **Table 3**. Slides were counterstained with Syto 40, washed with water and mounted with Citifluor AF1 (Citifluor Ltd., UK). Images were acquired using a Zeiss LSM700 (Carl Zeiss, Germany) with laser diode lines of 405 and 555 nm. Large images covering the entire granules were acquired using a 10×/0.45 plan-apochromat objective and high-resolution images were obtained with a 40×/1.3 plan-apochromat oil objective, using the averaging (*n* = 4) and tiling functions of Zeiss ZEN2010 software.

**Table 3:** Probes and hybridization conditions for FISH.

Probe	Target organism	FA^a^ (%)	Reference
Nse1472	*Nitrosomonas europaea/eutropha*	50	Juretschko et al., 1998
NEU^b^	*Nitrosomonas europaea/eutropha/halophila*	35	Wagner et al., 1995
Cluster6a192^b^	*Nitrosomonas oligotropha*	35	Adamczyk et al., 2003

*^a^FA = formamide concentration in hybridization buffer.*

*^b^Probe applied with unlabeled competitor probe according to the reference.*

## Results and Discussion

### Process Performance and Granulation

**FIGURE 1:**
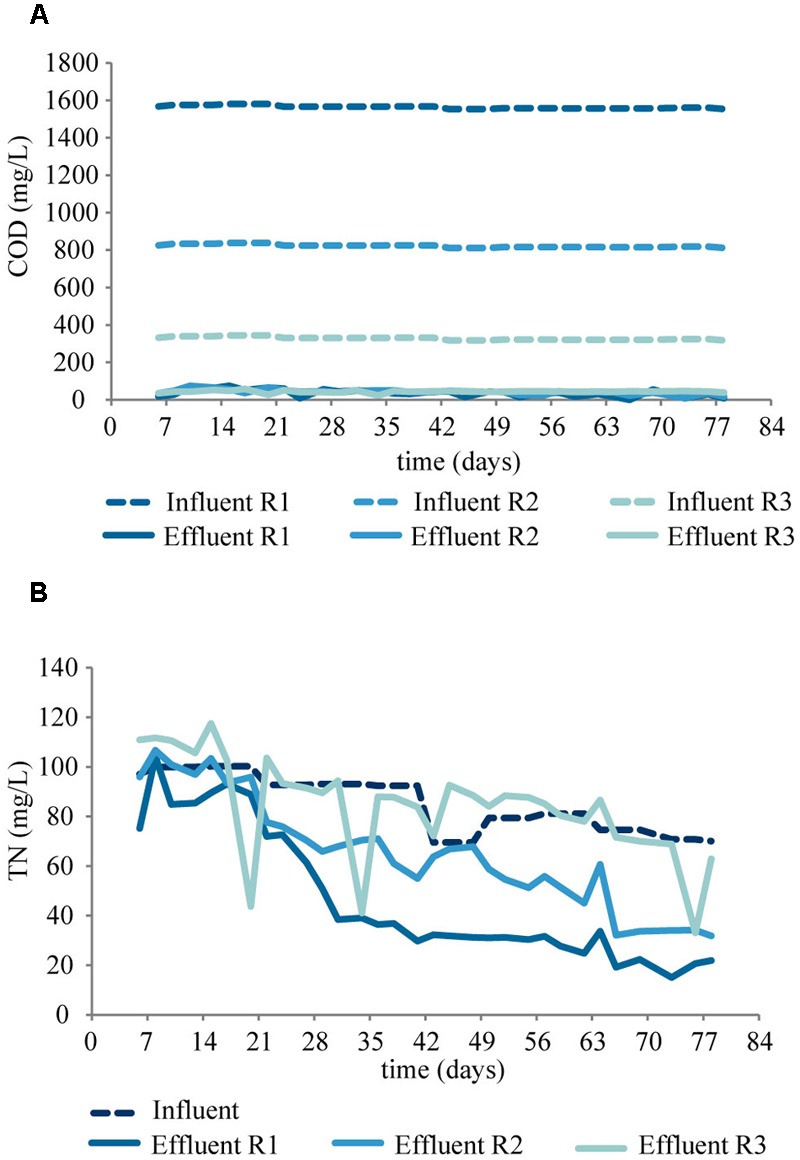
**COD (A)** and TN **(B)** concentration in the influents (dashed lines) and in the effluents (solid lines).

**FIGURE 2:**
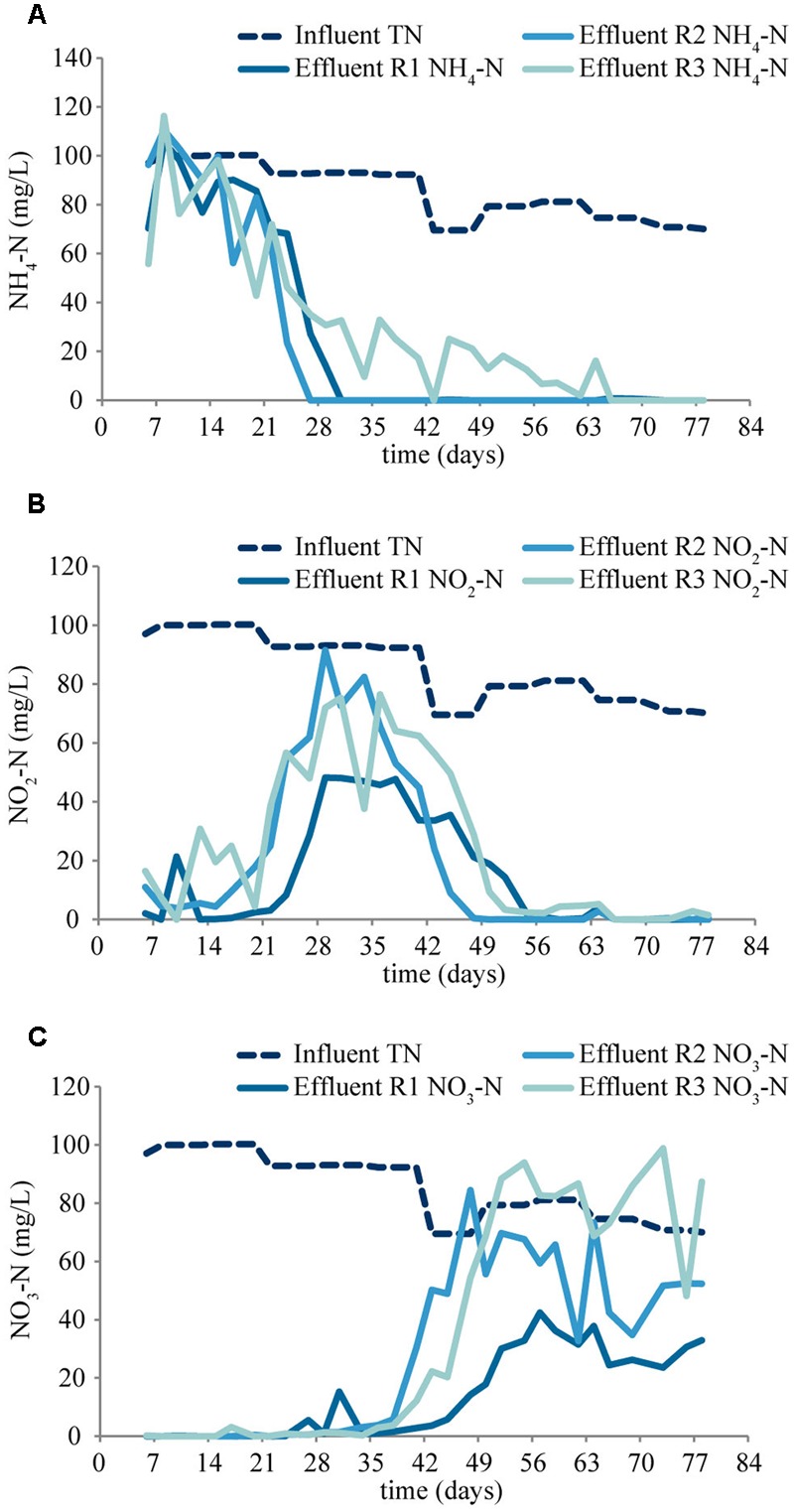
**Concentrations of NH_4_-N (A)**, NO_2_-N **(B)**, and NO_3_-N **(C)** in the effluents of the reactors.

**FIGURE 3:**
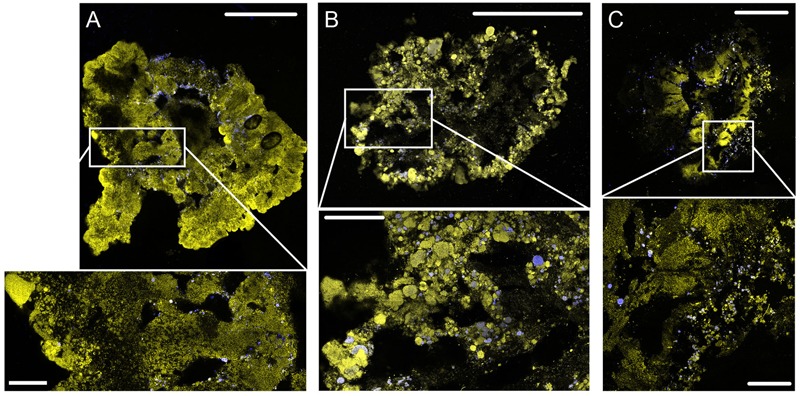
**FISH-CLSM images from cryosections of granules from R1 (A)**, R2 **(B)**, and R3 **(C)** at 200× magnification (upper images, scale bar: 500 μm) and detailed sections at 400× magnification (lower images, scale bar: 100 μm). Yellow: total cells (Syto 40); blue: AOB (Nse1472, NEU, Cluster6a192).

The process performance (COD and nitrogen concentrations) can be seen in **Figures 1, 2**. The COD removal was stable from the first day of operation, above 95, 90, and 80% in R1, R2, and R3, respectively (**Figure 1A**). Complete ammonium removal was achieved after 31 and 27 days in R1 and R2, while in R3 complete ammonium removal took longer time to establish (**Figure 2A**). The increase in nitrite concentrations until day 35 (**Figure 2B**) indicates an increasing activity of ammonia oxidizing bacteria (AOB). The subsequent decrease in nitrite concentrations, accompanied by an increase in nitrate concentration (**Figure 2C**) indicates an increasing activity of nitrite oxidizing bacteria (NOB). The process performance was considered stable from day 56. During the last 28 days of the experiment, the average ammonium removal was above 90% in all three reactors, and the average TN removal was 66, 38, and 0% in R1, R2, and R3, respectively (**Figure 1B**). The phosphorus concentration in the effluent was below detection limit (not shown). Granules started to form after approximately 7 days of operation. The granules were slightly smaller at lower OLR and at the end of the experiment the average diameters of the granules were 3.9 ± 1.5 mm, 3.6 ± 1.4 mm, and 3.3 ± 1.3 mm in R1, R2, and R3, respectively. FISH-CLSM images of granule cryosections showed a structure with channels and voids in the granules from all reactors (**Figure 3**). The biomass concentration followed the same pattern as the settled sludge height (Supplementary Figure S3) and stabilized at approximately 6, 2, and 1 g L^-1^ for R1, R2, and R3, respectively.

The observed succession of nitrite and nitrate accumulation was shown earlier (Pronk et al., 2015; Szabó et al., 2016). After the initial harsh wash-out conditions during start-up, the sludge started to granulate, and the better sludge retention allowed the increase of abundance and activity of AOB. The produced nitrite served then as substrate for NOB, resulting in increased NOB abundance and nitrate production (Val del Río et al., 2012; Szabó et al., 2016).

The COD:N ratio in R3 was 4.16 g COD/g N, which is near the theoretical limit where complete denitrification is possible, assuming a stoichiometric ratio of 4.2 g COD/g N (Carrera et al., 2004). However, as a result of competitive microbial processes, higher COD:N ratios are often required to reach full denitrification (Carrera et al., 2004; Choi et al., 2008). In granules, Guo et al. (2013) achieved approximately 60% TN removal in a continuously aerated reactor (DO < 1mg L^-1^) operated at 20°C with a COD:N ratio of 5. The complete lack of denitrification in reactor R3 was therefore unexpected, even though we did not anticipate full denitrification with the applied aeration strategy. To better understand the biochemical reactions during the anoxic and aerobic phases, cycle studies were performed.

### Carbon and Nitrogen Conversions within an SBR Cycle

#### Anoxic Phase

**FIGURE 4:**
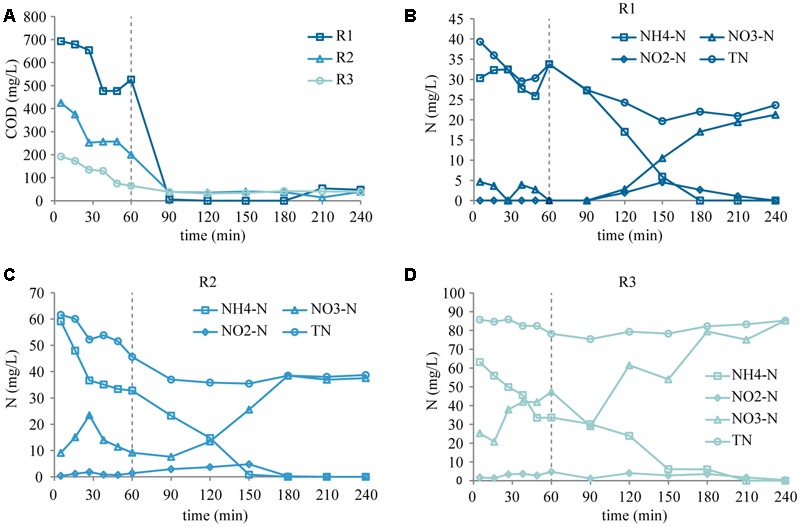
**COD (A)** and N concentration profiles during the cycle study in R1 **(B)**, R2 **(C)**, and R3 **(D)**. The dashed vertical line shows the transition between the anoxic and aerobic phase.

In the anoxic phase, COD was not completely consumed in any of the reactors (**Figure 4A**). The NH_4_-N concentration appeared to decrease during the anoxic phase in all three reactors. The nitrate concentration fluctuated, but was zero at the end of the phase in R1 (**Figure 4B**). In R2 the nitrate concentration appeared to increase in the first 30 min and subsequently decreased (**Figure 4C**), while in R3 it increased during the entire anoxic phase (**Figure 4D**). The patterns were partly a result of the diffusion limitations (below), partly due to residual oxygen from the previous cycle. Nonetheless, one can conclude that nitrate was consumed in R1, some nitrate consumption occurred also in R2, but probably not in R3. This indicates incomplete denitrification in R2, and the absence of denitrification in R3, in spite of the anoxic conditions.

The data obtained during the anoxic phase has to be interpreted with caution due to the lack of mixing and diffusion limitations. Before the anoxic phase, the fresh influent was pulse-fed through the stagnant sludge bed from the bottom of the reactors. This creates a strong substrate gradient and allows the substrate to penetrate the granules, and therefore pulse feeding through stagnant bed has become a common practice within the field (Li et al., 2014; Cydzik-Kwiatkowska, 2015; Zou et al., 2015). However, the lack of mixing posed a challenge for sampling during the anoxic phase of the cycle study. The sampling point was located in the lower half of the reactor, just above the sludge bed. Diffusion, to even out the concentration differences between the fresh feed and the remaining water from the previous cycle (57% of the reactor volume), may have taken place most of the time of the anoxic phase (see Supplementary Figure S2 for empty bed diffusion patterns), which is assumed to be the main reason behind the decreasing ammonium and increasing nitrate concentrations. Ammonium could also be removed by adsorption to the biomass (Bassin et al., 2011), while anammox was likely not a major contributor to the decrease in ammonium concentrations in this phase, since nitrite concentrations were low and unaltered.

The residual oxygen from the previous cycle is estimated to be 2 mg L^-1^ at the beginning of the cycle, and increased to maximum 5 mg L^-1^ due to diffusion. It was depleted within 20 min, presumably by heterotrophs, due to their competitive advantage over AOBs, while maximum 1–3% of the influent COD was oxidized. Therefore, sufficient COD was available for denitrification at the beginning of the anoxic phase in every reactor, and at the end of the anoxic phase the COD concentrations were still considerable. This suggests that either the anoxic phase was not long enough (in R2), or nitrate was limiting (in case of R1). In R3, the absence of nitrate consumption and the decrease in COD concentration indicates that the organic matter was consumed through another anaerobic metabolic pathway, presumably polyhydroxyalkanoate (PHA) storage (Third et al., 2003; Qin et al., 2005). Increasing the length of the anoxic phase or mixing the reactor content would probably have increased the denitrification efficiency in R2, and possibly even in R3, while in cases like R1 intermittent aeration could increase the nitrate availability (Chen et al., 2013; Pan et al., 2013; Zhong et al., 2013) and facilitate denitrification.

#### Aerobic Phase

In the aerobic phase (**Figure 4**), the residual COD was rapidly consumed in all three reactors (within 30 min). NH_4_-N was also completely removed in all three reactors (within 90–120 min), via autotrophic nitrification and heterotrophic assimilation. All nitrite produced was converted to nitrate by the end of the aerobic phase.

In R1 and R2, the produced NO_3_-N was 21 and 38 mg N L^-1^ per cycle, respectively, less than the consumed NH_4_-N (85 mg N L^-1^ per cycle). This suggests that some nitrate was removed by denitrification in the anoxic core of the granule, and even anaerobic ammonium oxidation (anammox) may have occurred. Alternatively, ammonium was assimilated by heterotrophs at the surface of the granule. Due to the moderate biomass production observed in the reactors (Supplementary Figure S3) it can be assumed that only approximately 33% (R1) and 15% (R2) of the influent ammonium was assimilated, and approximately 33% (R1) and 23% (R2) was removed via simultaneous nitrification–denitrification. Studies on oxygen diffusion (with saturated DO concentration in the bulk) show that the penetration depth of oxygen can vary between 100 and 900 μm in 2–3 mm granules, depending on the bulk concentration of organic matter and ammonium (Li and Liu, 2005; Su and Yu, 2005; Chiu et al., 2007; de Kreuk et al., 2007). The average diameters of our granules were 3.9 ± 1.5 mm, 3.6 ± 1.4 mm, and 3.3 ± 1.3 mm in R1, R2, and R3, respectively, which are large enough to enable anoxic metabolic activities in the core.

In spite of the large granule size and the availability of organic matter and nitrate, nitrogen removal via simultaneous denitrification or anammox was not observed in R3. A tentative explanation is that oxygen penetrated the deeper region of the granules through channels in the biomass, which would prevent denitrification as well as anammox. FISH-CLSM images of intact granule cryosections from R3 (**Figure 3C**) show the presence of channels and voids in the core of the granule. Aerobic AOB were found along these channels and voids, indicating the presence of oxygen in these areas. However, similar structures were found in granules from R1 and R2 (**Figures 3A,B**). Therefore, the lack of simultaneous denitrification in R3 is assumed to originate from multiple factors, namely oxygen penetration through channels, low COD loading rate and possibly also competition for organic matter between denitrifying and PHA producing bacteria.

### Alpha-Diversity and Microbial Community Dynamics

**FIGURE 5:**
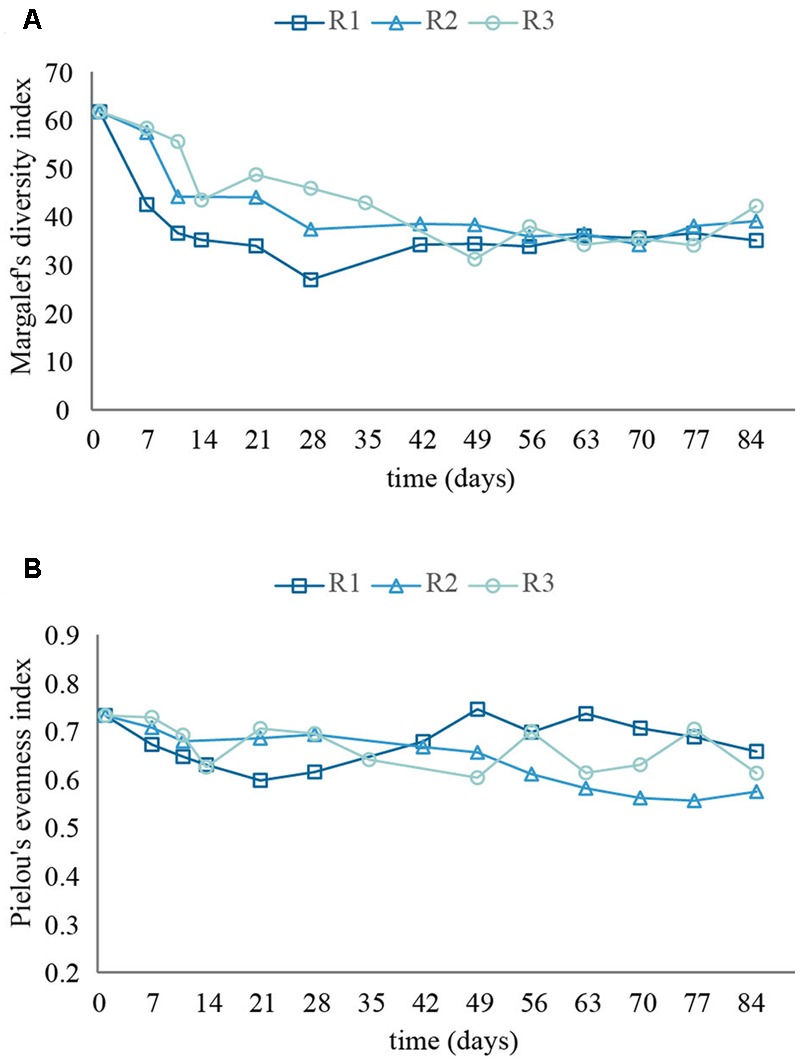
**Richness (A)** and evenness **(B)** of the microbial communities in the reactors.

During the start-up period, the richness decreased in all three reactors (**Figure 5A**). The reactor with the highest load experienced the steepest decrease in richness. By the end of the experiment all three reactors showed similar richness, approximately 37% lower than the initial richness of the seed sludge. The evenness (**Figure 5B**) decreased in R1 during start-up, but recovered during steady-state operation. In R2, the main decrease of evenness occurred in the later phase of the study, while in R3, evenness fluctuated throughout the whole experiment. At the end of the experiment, all three reactors showed an evenness around 16% lower than that of the seed sludge.

**FIGURE 6:**
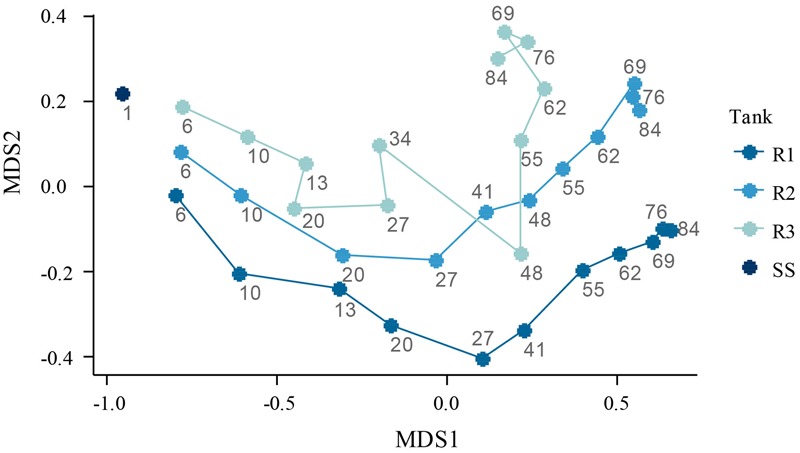
**NMDS ordination of the microbial communities in the reactors (R1–R3) and in the seed sludge (SS).** The numbers in the plot refer to days of reactor operation. Stress = 0.07.

The microbial population dynamics was visualized with the help of NMDS ordination (**Figure 6**). In spite of the common seed sludge, the reactors’ microbiomes showed less than 50% similarity already after 6 days, and the dissimilarity continued to increase. By the end of the experiment, the dissimilarity between R1 and R2 was ca. 60%, and between R2 and R3 ca. 62%. The largest dissimilarity (74%) was observed between R1 and R3, the reactors fed with the highest and lowest OLR.

The decreased diversity in the lab-scale reactors compared to the seed sludge is presumably caused by the lack of complex substrates. Acetate- (and propionate-) fed granular sludge has earlier been reported to develop dominant-species-based communities with low richness (Gonzalez-Gil and Holliger, 2011). By contrast, pilot-scale granular sludge reactors fed with real wastewater have been reported to show similar diversity as full-scale floccular activated sludge (Winkler et al., 2013). The population in R1 (the reactor with the highest load) adjusted most rapidly to the changed substrate conditions, which explains why diversity decreased faster in this reactor. Similar trends of diversity being dependent on the organic load during granulation have been shown for glucose-fed aerobic granular reactors (Li et al., 2008).

The large difference between the composition of the communities early in the experiment could be a result of the highly dynamic conditions and stochastic processes. However, microbial communities in activated sludge flocs have shown significant similarity between replicate reactors at all times (Ayarza et al., 2010), which suggests that the rapidly diverging communities in our reactors were a result of the different operational conditions (i.e., deterministic factors).

### Identity and Dynamics of Major Taxa in the Granular Microbiomes

The temporal variation of the most abundant phyla in the reactors is shown in Supplementary Figure S4. *Proteobacteria* and *Bacteroidetes* were the most abundant phyla in all three reactors, adding up to 90% of the total read abundance on average. This is in agreement with earlier published reports about granular sludge and conventional activated sludge. However, identification of the bacterial community on phylum- (or even family-) level gives very little information about the possible functions of the microbiome (McIlroy et al., 2015). Using NGS methods and a taxonomic database (MiDAS) that allows genus-level classification we can discuss the putative functions of the bacterial community in the granules. To allow better comparison with earlier granular sludge studies, the most abundant families are shown in Supplementary Figure S4.

**FIGURE 7:**
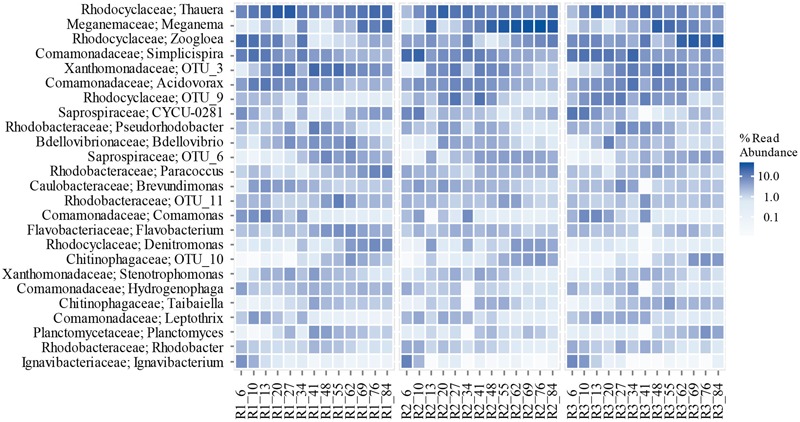
**Temporal variation of the most abundant genera in the reactors.** The labels on the *x*-axis refer to the reactor and the days of operation since start-up. (OTU_3, OTU_6, OTU_9, OTU_10, and OTU_11 were unclassified on the genus level.)

The most abundant genera observed in our reactors are shown in **Figure 7**. By the end of the experiment, *Meganema* (19%), *Thauera* (17%), and *Paracoccus* (8%) dominated the reactor with the highest OLR (R1), adding up to 44% of the read abundance. In R2, *Meganema* (34%), *Thauera* (12%), and *Zoogloea* (9%) were the most abundant genera, while in the reactor with the lowest OLR (R3) *Zoogloea* (33%) and *Thauera* (6%) were most abundant. At the end of the experiment, the relative read abundance of *Thauera* was highest in the reactor with highest OLR, and lowest at the lowest OLR. *Zoogloea* showed opposite correlation: its abundance was highest at lowest OLR. *Meganema* was most abundant in R2, followed by R1 and then R3. *Paracoccus* was dominant only in R1.

These four genera have earlier been reported in different granular sludge reactors operated for organic carbon and nitrogen removal (Li et al., 2008; Ebrahimi et al., 2010; Liu et al., 2010; Zhao et al., 2013, 2015; Kong et al., 2014; Lv et al., 2014; Cydzik-Kwiatkowska, 2015; Figueroa et al., 2015). *Thauera* and *Zoogloea* have been found at both low and high OLRs, between 1 and 15 kg COD m^-3^d^-1^ (Ebrahimi et al., 2010; Zhao et al., 2013; Lv et al., 2014). *Meganema* has been found to be abundant in reactors operated at 1.5–3 kg COD m^-3^d^-1^, treating industrial or synthetic wastewater (Kong et al., 2014; Figueroa et al., 2015). *Paracoccus* has been reported at loading rates of 1.5–3.3 kg COD m^-3^d^-1^, in reactors treating synthetic wastewater, and in wastewater polluted with pharmaceuticals (Lv et al., 2014; Cydzik-Kwiatkowska, 2015; Zhao et al., 2015). The roles of these and other contributors in the microbial communities are discussed below (see section Composition and Diversity of Functional Groups).

A detailed illustration of the temporal variation of each of the most abundant genera is shown in Supplementary Figure S5. As can be seen, the relative read abundance of the most abundant genera changed considerably even during the last 28 days, when the process performance was at steady state. This dynamic behavior cannot be observed when the population is analyzed in one single sample, and can lead to false conclusions about correlations between operational parameters and the dominant taxa. On the other hand, the identity of the core community seems to be relatively stable, even if the proportions of these taxa change with time during steady-state operation (28 days in this experiment).

### Composition and Diversity of Functional Groups

Even though the different loading rates selected for bacterial communities dominated by different genera, these genera could be assigned similar roles in the bacterial ecosystem of extracellular polymeric substances (EPS) production, denitrification, and PHA storage (Larsen et al., 2008; Oshiki et al., 2008; McIlroy et al., 2015, 2016). Also, the most abundant taxa did not account for an important function of the reactors, nitrification. It has been shown before that rare taxa can be disproportionately active (Power et al., 1996; Shade and Handelsman, 2012), therefore, it is important to assess the membership of the different functional groups, and not only the identity of the most abundant taxa.

Functional groups usually consist of many (phylogenetically related or unrelated) bacteria, and in case of decreasing diversity species may be compelled into keystone roles (Power et al., 1996), while a functionally redundant microbiome may enable the system to maintain its function even in case of disturbances (Briones and Raskin, 2003; Shade and Handelsman, 2012). Studies have demonstrated the correlation between microbial diversity and bioreactor stability both in laboratory-scale and full-scale reactors treating wastewater (e.g., von Canstein et al., 2002; Yang et al., 2011). In the following sections, we discuss important ecosystem functions in our granular sludge bioreactors: EPS production, hydrolysis, nitrification, denitrification, and the uptake of organic carbon (Weissbrodt et al., 2014).

#### EPS Production

**FIGURE 8:**
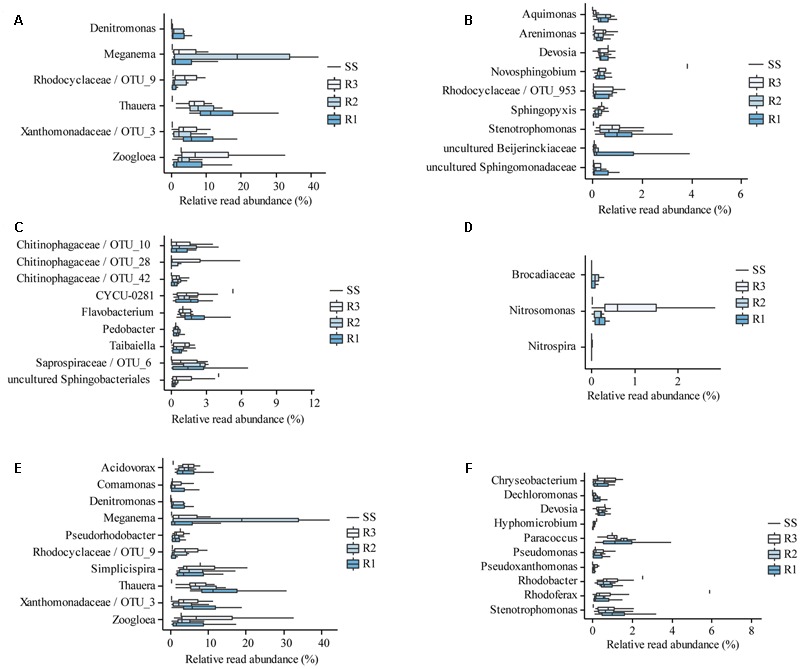
**Composition and diversity of functional groups.** Abundant **(A)** and rare **(B)** EPS producing taxa; hydrolyzing taxa **(C)**; nitrifying bacteria **(D)**; abundant **(E)**; and rare **(F)** denitrifying taxa in the reactors (R1–R3) and the SS. The bounds of the boxes show the first and third quartile, and the band inside the boxes shows the second quartile (median). The whiskers extend to the most extreme point that is within 1.5-times the interquartile range. N.B. “*Denitromonas*” is not yet an established genus of its own, although it is present in many databases. The closest established genus is *Azoarcus*.

The EPS producers (**Figure 8A**) comprise one of the most abundant functional groups in all three reactors (approximately 40% on average), while it’s cumulative relative read abundance added up only to approximately 13% in the seed sludge. Besides the most abundant genera [*Meganema, Thauera*, and *Zoogloea* (Larsen et al., 2008)], many less abundant but persistent taxa were found that are putative EPS producers (**Figure 8B**). Little is known about the physiological characteristics of these genera, but the families *Rhodocyclaceae, Xanthomonadaceae, Sphingomonadaceae, Beijerinckiaceae*, and *Hyphomicrobiaceae* are reported to include a variety of EPS producing bacteria (Song et al., 2013; Rosenberg et al., 2014; Weissbrodt et al., 2014).

Selection for EPS producing bacteria in granular reactors seems reasonable, since EPS is a key component of biofilms, thus it plays an important role in granule formation and (mechanical) stability (Weber et al., 2007; Lemaire et al., 2008a; Tan et al., 2014).

#### Hydrolysis

In the seed sludge, the *Saprospiraceae* related genus CYCU-0281 (McIlroy et al., 2015) and an uncultured *Sphingobacteriaceae* related genus were found to be the most abundant hydrolyzing bacteria (**Figure 8C**). In the laboratory scale reactors a more diverse hydrolyzing community could be observed: four *Chitinophagaceae* related genera (including *Taibaiella*), *Pedobacter* (Steyn et al., 1998), and an unclassified *Saprospiraceae* related genera. Members of the order *Sphingobacteriales* (Nielsen et al., 2010; Rosenberg et al., 2014), and species of *Flavobacterium* were also found in our reactors, which have been reported to hydrolyze various substrates (Bernardet et al., 1996; Nielsen et al., 2010; Rosenberg et al., 2014).

In real domestic wastewater, large part of the organic carbon is present as complex compounds like proteins, lipids, and polysaccharides, the substrates for hydrolyzing bacteria (Nielsen et al., 2010). In our laboratory-scale reactors, acetate was the main source of carbon, but the relative read abundance of hydrolyzing bacteria in the granular sludge was similar to that in the seed sludge (approximately 10%). It is likely that these bacteria utilized soluble microbial products, dead biomass and EPS (Kindaichi et al., 2004; Okabe et al., 2005) and suggests a considerable turnover of organic carbon within the granules.

#### Nitrification

Although the nitrite and nitrate concentration profiles (**Figures 2, 4**) clearly indicate that nitrifying bacteria have a notable contribution to the nitrogen conversion in all three reactors, the relative read abundances of AOB and NOB were low (**Figure 8D** and Supplementary Figure S6). However, it was shown before that nitrification can be the main pathway for ammonium removal even at low relative abundances of AOB and NOB (Szabó et al., 2016). Furthermore, AOB were detected by FISH-CLSM in all three reactors along the biomass-water interfaces (including channels) where the availability of oxygen is highest (**Figure 3**). The relative read abundance of AOB (*Nitrosomonas*) was higher in R3, presumably due to less heterotrophic growth at the lower OLR in this reactor.

Only few *Nitrospira* and no *Nitrobacter* was detected in the reactors. *Nitrobacter* has previously been reported to be the dominant NOB in another acetate-fed granular sludge reactor (e.g., Winkler et al., 2012). However, *Nitrospira* is generally favored by low substrate concentrations (Kim and Kim, 2006), which may explain their occurrence in the reactors at stable operation, when nitrite concentrations were low (**Figure 2**). *Nitrospira* has earlier been found to be the dominant NOB in laboratory-scale reactors operated at low nitrogen concentrations (Meyer et al., 2005; Lemaire et al., 2008b; Weissbrodt et al., 2014).

Anammox bacteria were also detected, at low relative read abundances. In particular, bacteria within *Brocadiaceae* were observed in R1 and R2, suggesting that anammox may have contributed to the observed nitrogen removal in these reactors. Coexistence of communities of AOB, NOB, anammox, and denitrifying bacteria in granular SBRs is possible due to the different microenvironments and operational phases (Langone et al., 2014).

#### Denitrification

Many of the EPS producing genera found in our reactors are also considered to be denitrifiers (**Figures 8E,F**), e.g., *Denitromonas, Meganema, Thauera, Devosia*, and *Stenotrophomonas* (Etchebehere et al., 2003; Lu et al., 2014; McIlroy et al., 2015, 2016). The group of denitrifiers was large and versatile, with at least 20 different taxa in our reactors. The cumulative relative read abundance of denitrifiers was 25% in the seed sludge, and 45–60% at the end of the experiment in the three reactors. Most of these genera are mixotrophic bacteria, and not all of them are necessarily denitrifying. Since denitrification was not observed in R3, it can be assumed that the diverse and abundant denitrifying population in R3 was actually sustained through other metabolic pathways (e.g., aerobic respiration).

#### Uptake and Storage of Organic Carbon

Organic carbon can be removed from wastewater through different metabolic pathways, for example assimilation, aerobic respiration, denitrification, EPS, and PHA production. The competition between these different processes strongly influence the bacterial population. The alternating anaerobic (anoxic) and aerobic operation strategy applied in our reactors selects for mixotrophic bacteria (e.g., denitrifiers), while the anaerobic pulse feeding favors bacteria that can accumulate storage polymers. It is estimated that 75% of the activated sludge bacteria are capable of producing storage polymers (Inoue et al., 2016), for example *Acidovorax, Chryseobacterium, Comamonas, Dechloromonas, Flavobacterium, Meganema, Paracoccus, Rhodoferax, Simplicispira, Sphingopyxis, Thauera*, and *Zoogloea* (Kragelund et al., 2005; Oshiki et al., 2008; McIlroy et al., 2015; Inoue et al., 2016). Many denitrifying bacteria can produce PHA, and our results suggest that the operating conditions in R3 may have favored PHA production over denitrification.

## Conclusion

Three laboratory-SBRs were operated for 12 weeks to study the impact of OLRs on the community assembly of anoxic/aerobic granular sludge. In-depth analysis of the bacterial communities, by high-throughput amplicon sequencing, was performed to investigate the interplay between the microbial community structure and the performance and stability of granular sludge reactors.

Granules of similar sizes developed at all OLRs. Complete COD- and ammonium removal was achieved in all three reactors, but the average TN removal was only 66, 38, and 0% in R1, R2, and R3, respectively. Cycle studies and FISH-CLSM of cryosections suggested anoxic time, nitrate availability and oxygen penetration in the granules via channels as important factors for the nitrogen removal.

Community diversity decreased in all three reactors during start-up, presumably due to the lack of complex substrates in the influent, with largest change for the reactor with the highest load. In spite of the differences during start-up, all three reactors showed similar richness and evenness as granules formed and the reactors reached steady state operation.

The bacterial communities at different loading rates diverged rapidly after start-up, showing less than 40% similarity by the end of the experiment.

Different genera dominated the bacterial communities at the different loading rates, but these genera have similar roles, namely EPS production, denitrification, and PHA storage. EPS plays an important role in granule formation, the alternating anoxic–aerobic strategy selects for mixotrophic bacteria, and the anaerobic pulse feeding favors bacteria with storage capability. Many less abundant but persistent taxa were also detected in every functional group (except the group of nitrifying bacteria).

Both the dominant and less abundant community members showed considerable temporal dynamics even at steady state reactor conditions. This suggests that functionally redundant microbiomes assembled during granulation, irrespective of the OLRs.

## Author Contributions

ES, FP, and B-MW conceived and designed the experiments; ES, RL, and FP contributed to the acquisition of the data; ES, RL, and FP contributed to the analysis of the data; ES, RL, MH, OM, FP, and B-MW contributed to the interpretation of the data.

## Conflict of Interest Statement

The authors declare that the research was conducted in the absence of any commercial or financial relationships that could be construed as a potential conflict of interest.
