# Supplementary material for: Microbial Population Dynamics and Ecosystem Functions of Anoxic/Aerobic Granular Sludge in Sequencing Batch Reactors Operated at Different Organic Loading Rates
Source: Front Microbiol. 2017 May 1;8:770. doi: 10.3389/fmicb.2017.00770 (PMC5410608; doi:10.3389/fmicb.2017.00770)
Supplement: Supplementary file 1 [file Image_1.pdf]

Supplementary Material

Microbial population dynamics and ecosystem functions of aerobic granular sludge in sequencing batch reactors operated at different organic loading rates

Enikö Szabó[1,*], Raquel Liébana[1], Malte Hermansson[2], Oskar Modin[1], Frank Persson[1], Britt-Marie Wilén[1]

[1] Division of Water Environment Technology, Department of Civil and Environmental Engineering, Chalmers University of Technology, Gothenburg, Sweden

[2] Department of Chemistry and Molecular Biology, University of Gothenburg, Gothenburg, Sweden

*** Correspondence:** Enikö Szabó eniko.szabo@chalmers.se

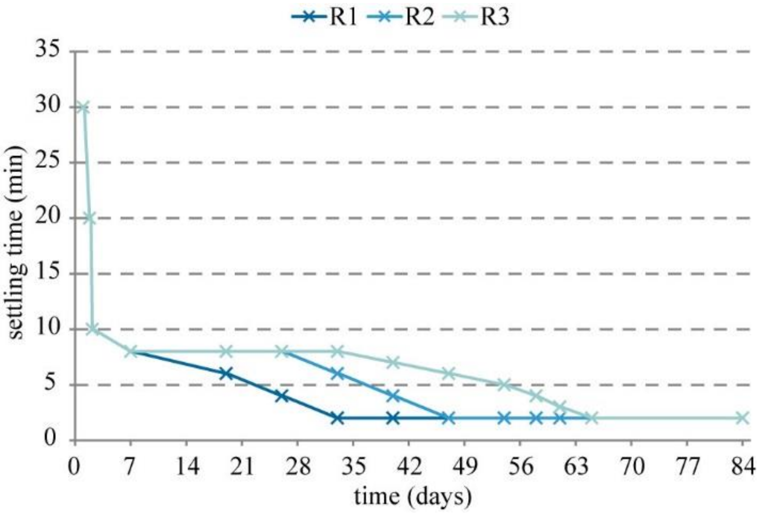

Supplementary Figure 1. Gradually decreased settling time

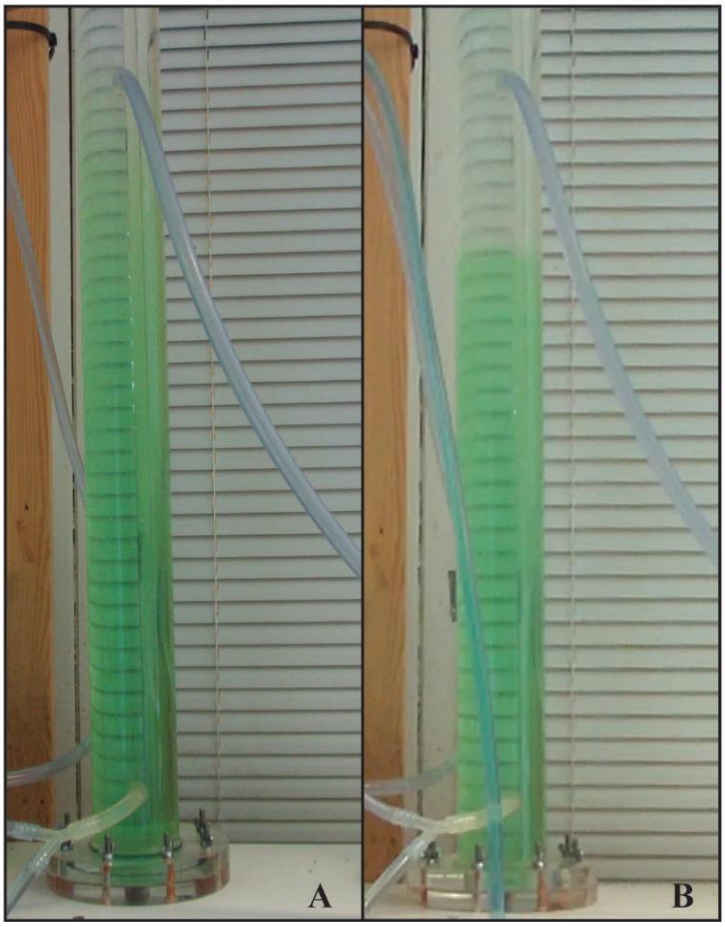

Supplementary Figure 2. Demonstration of the hydrodynamic conditions during the anoxic phase (with stained influent, and without sludge, for better visibility). Due to the plug-flow influent, the fresh wastewater remains at the bottom half of the reactor after feeding (**A**, 5 min). The effect of diffusion dilutes the fresh influent, but the substrate concentration is still high at the end of the pre-anoxic phase (**B**, 60 min). Green = fresh influent, colorless = residual liquid from the previous cycle. N.B. during the experiment, the stagnant sludge bed influenced the hydrodynamic conditions, e.g. the sludge partly displaced the influent due to the volume of the biomass and the volume of the residual liquid that fills the voids and pores of the granules.

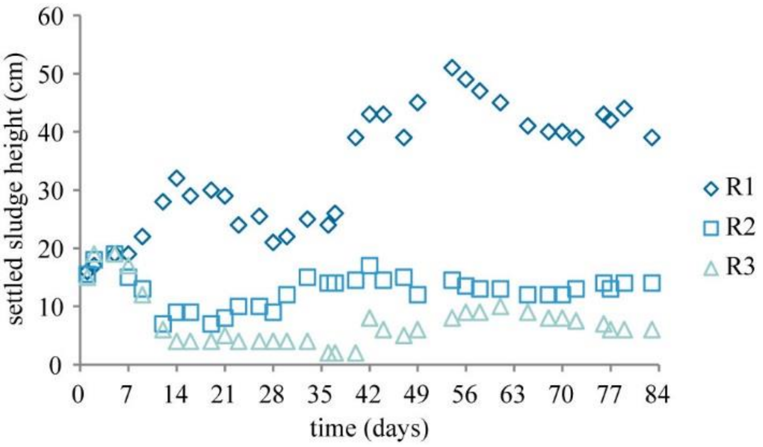

Supplementary Figure 3. Settled sludge height in the reactors

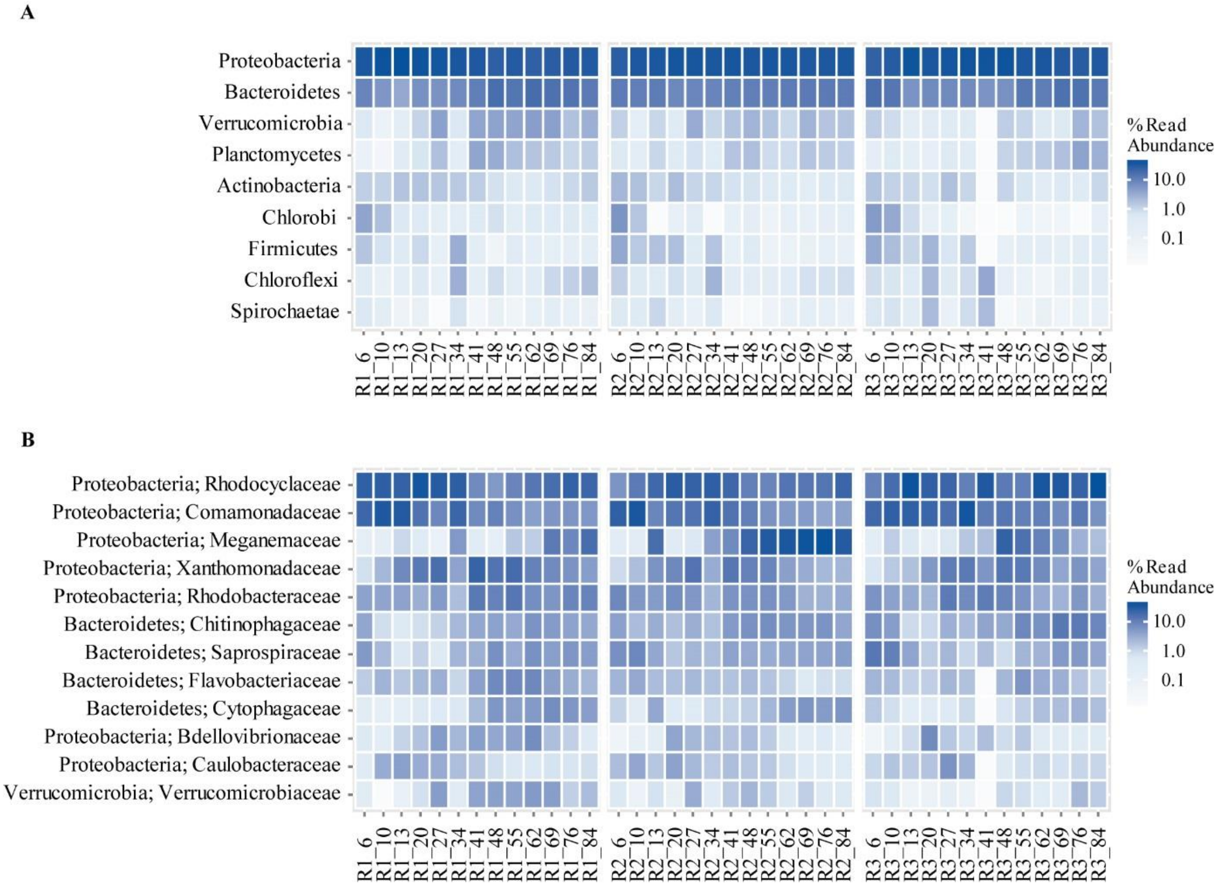

Supplementary Figure 4. Temporal variation of the most abundant phyla (**A**) and families (**B**) in the reactors. The labels on the x-axis refer to the reactor and the days of operation since start-up

Supplementary Figure 5. Temporal variation in the relative read abundance of the most abundant genera. (OTU_3, OTU_6, OTU_9, OTU_10 and OTU_11 were unclassified on the genus level.)

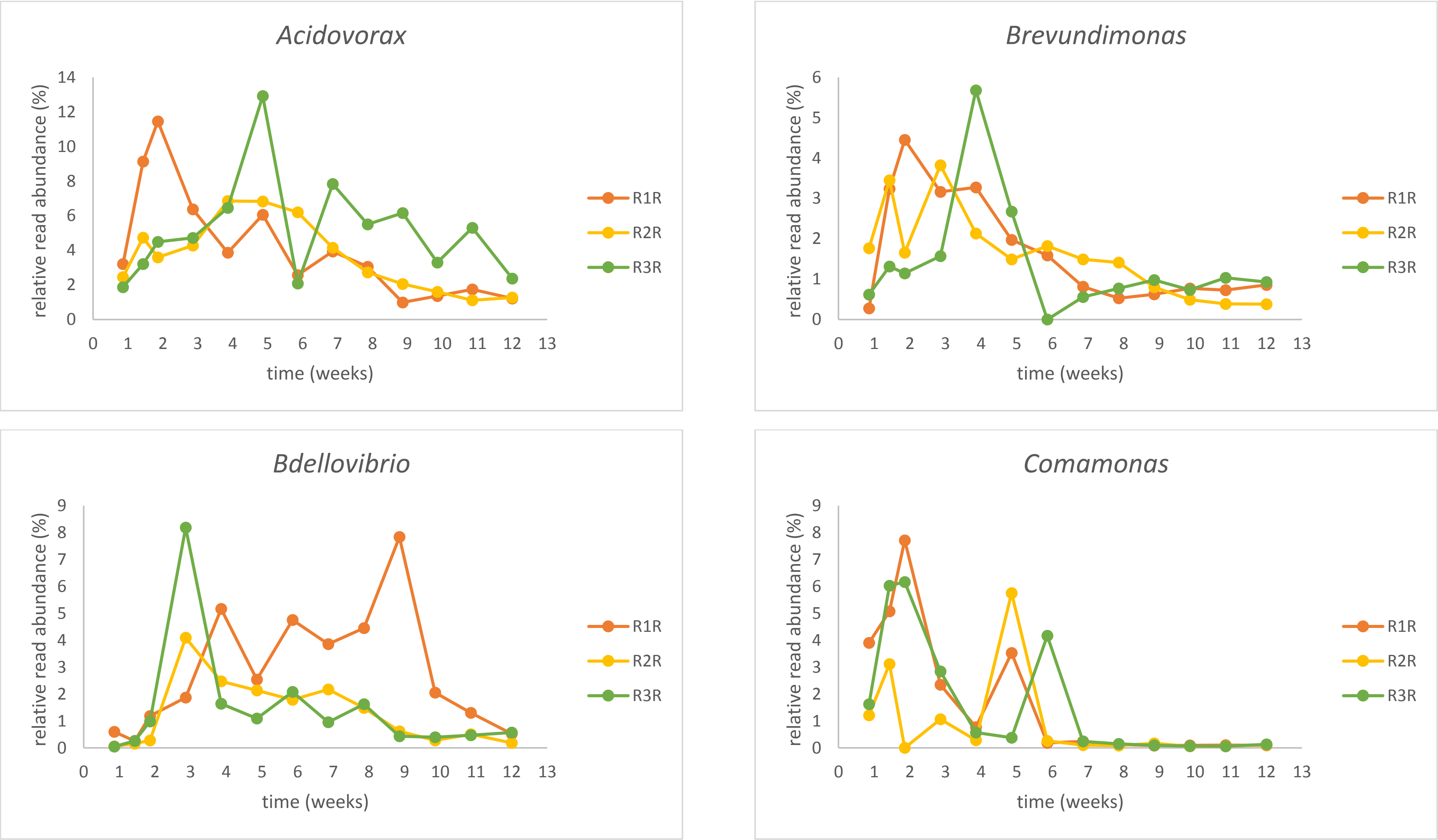

Supplementary Figure 5. - continued.

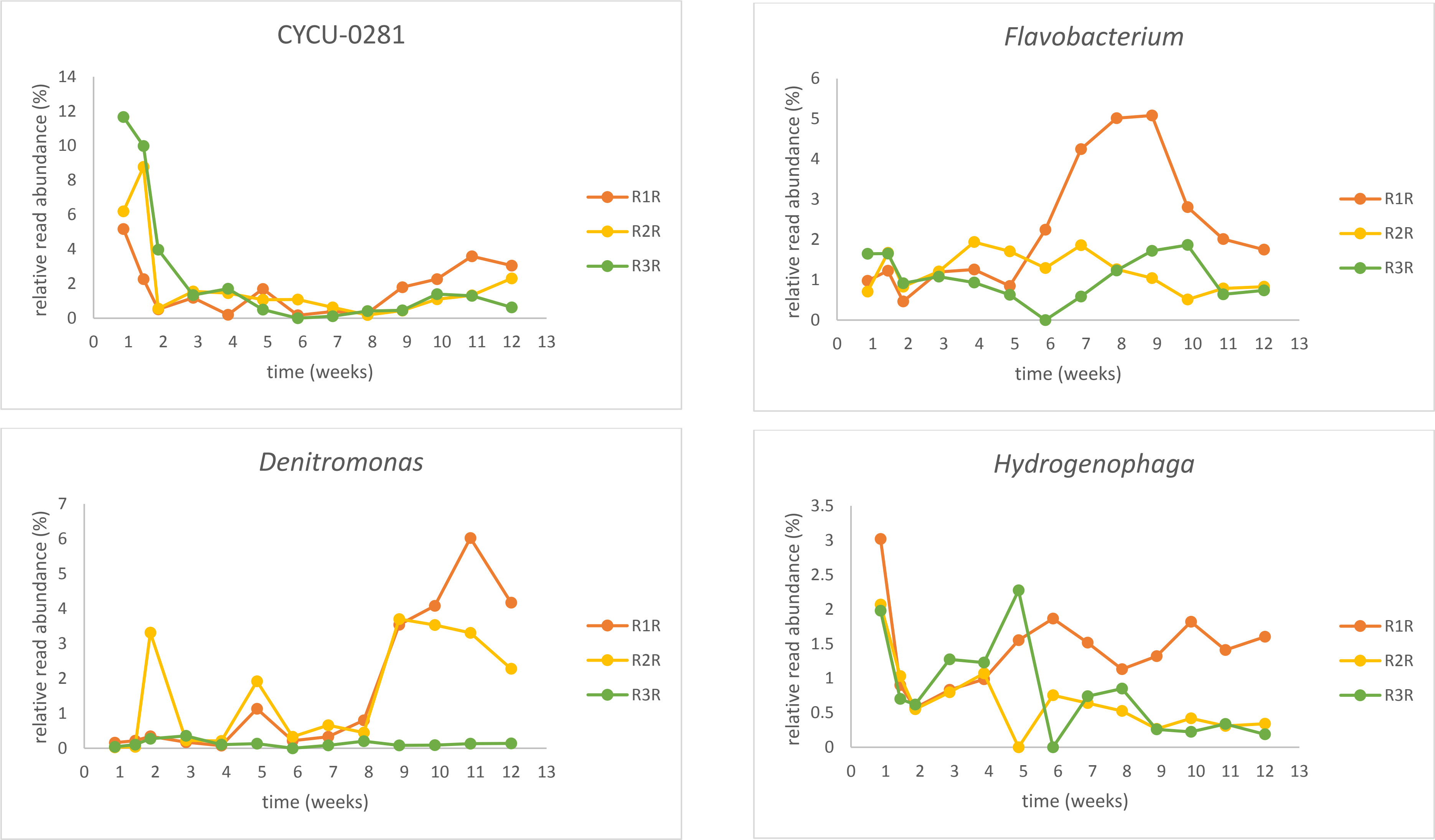

Supplementary Figure 5. - continued.

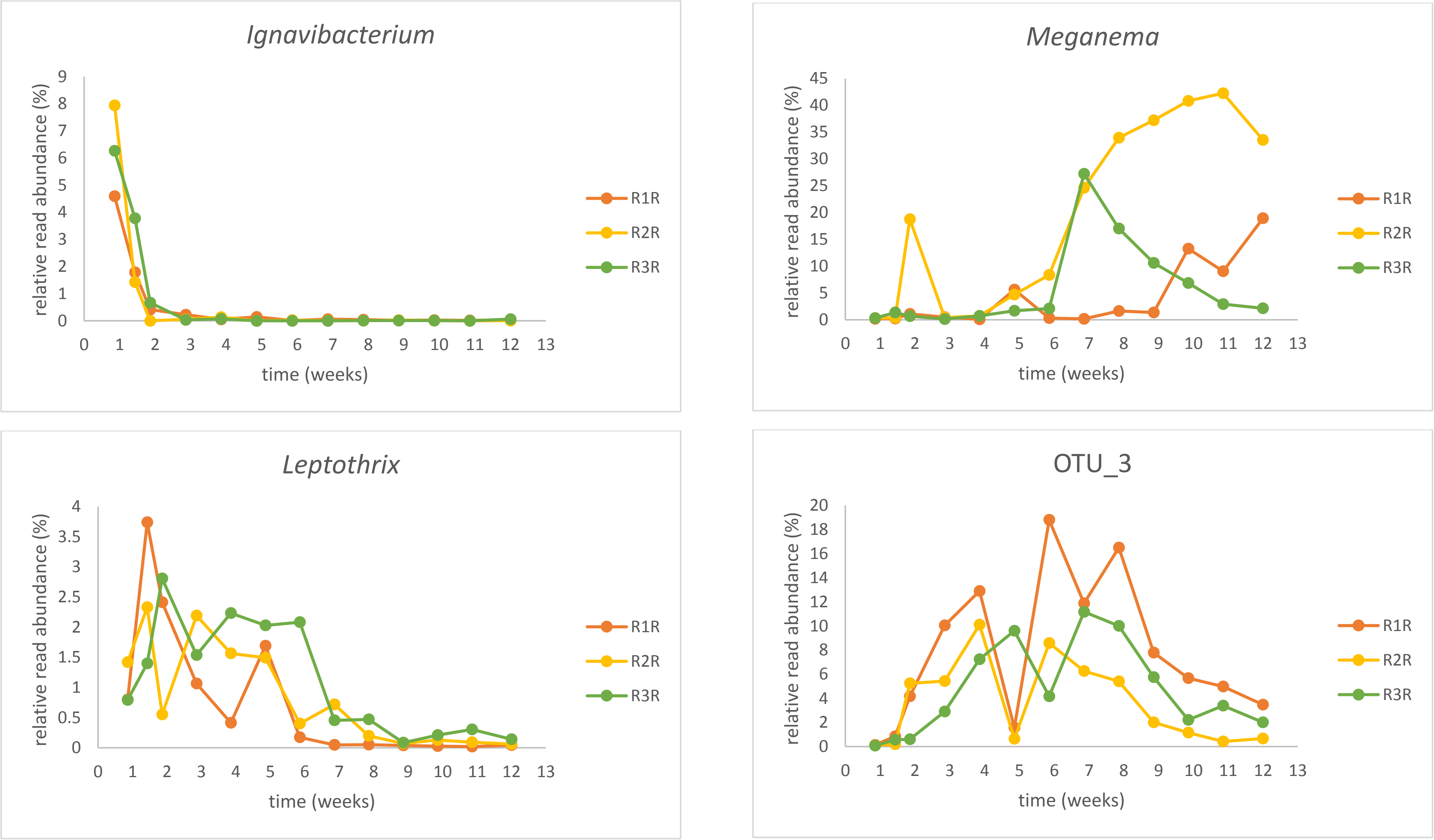

Supplementary Figure 5. - continued

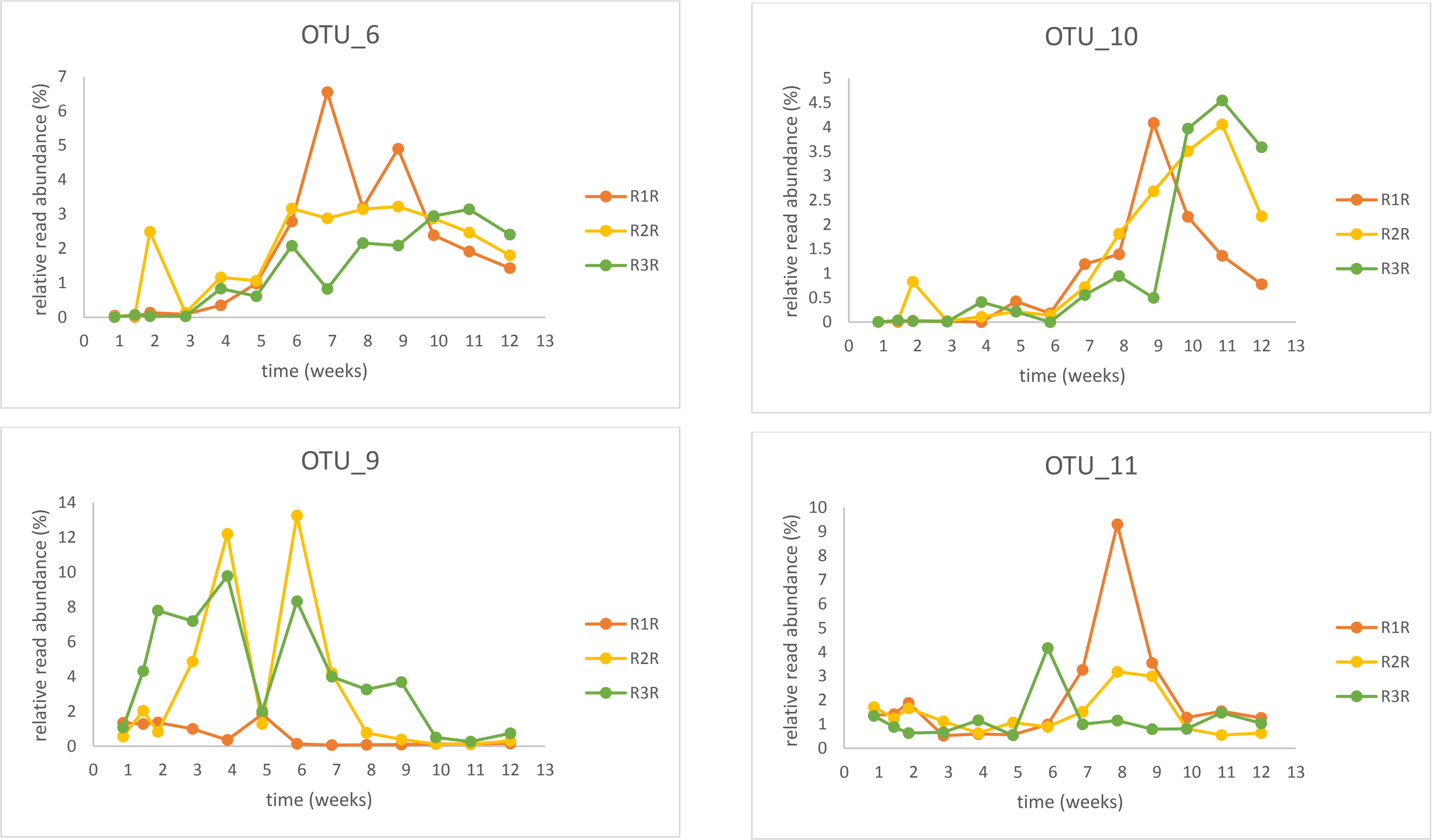

Supplementary Figure 5. - continued

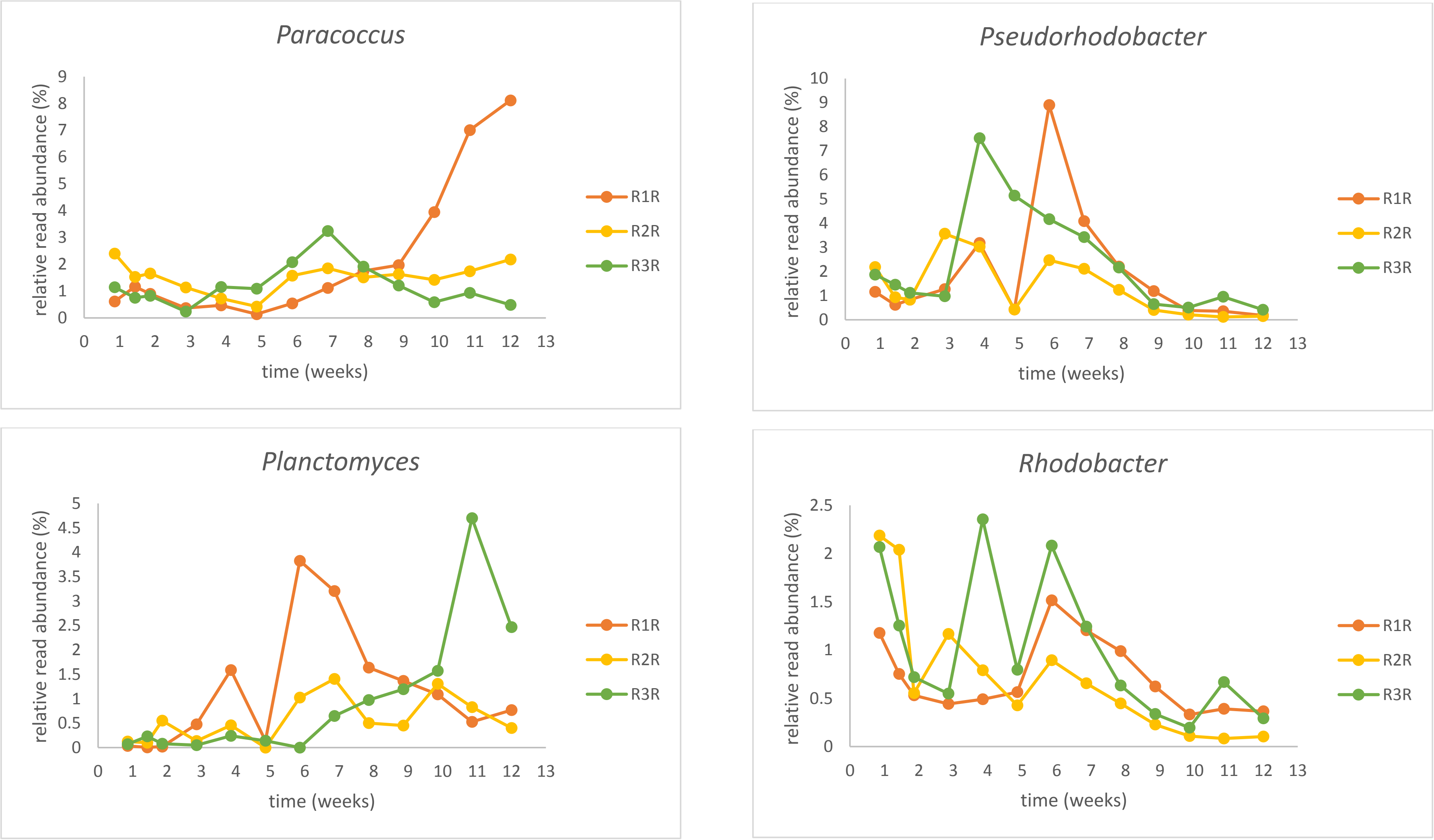

Supplementary Figure 5. - continued

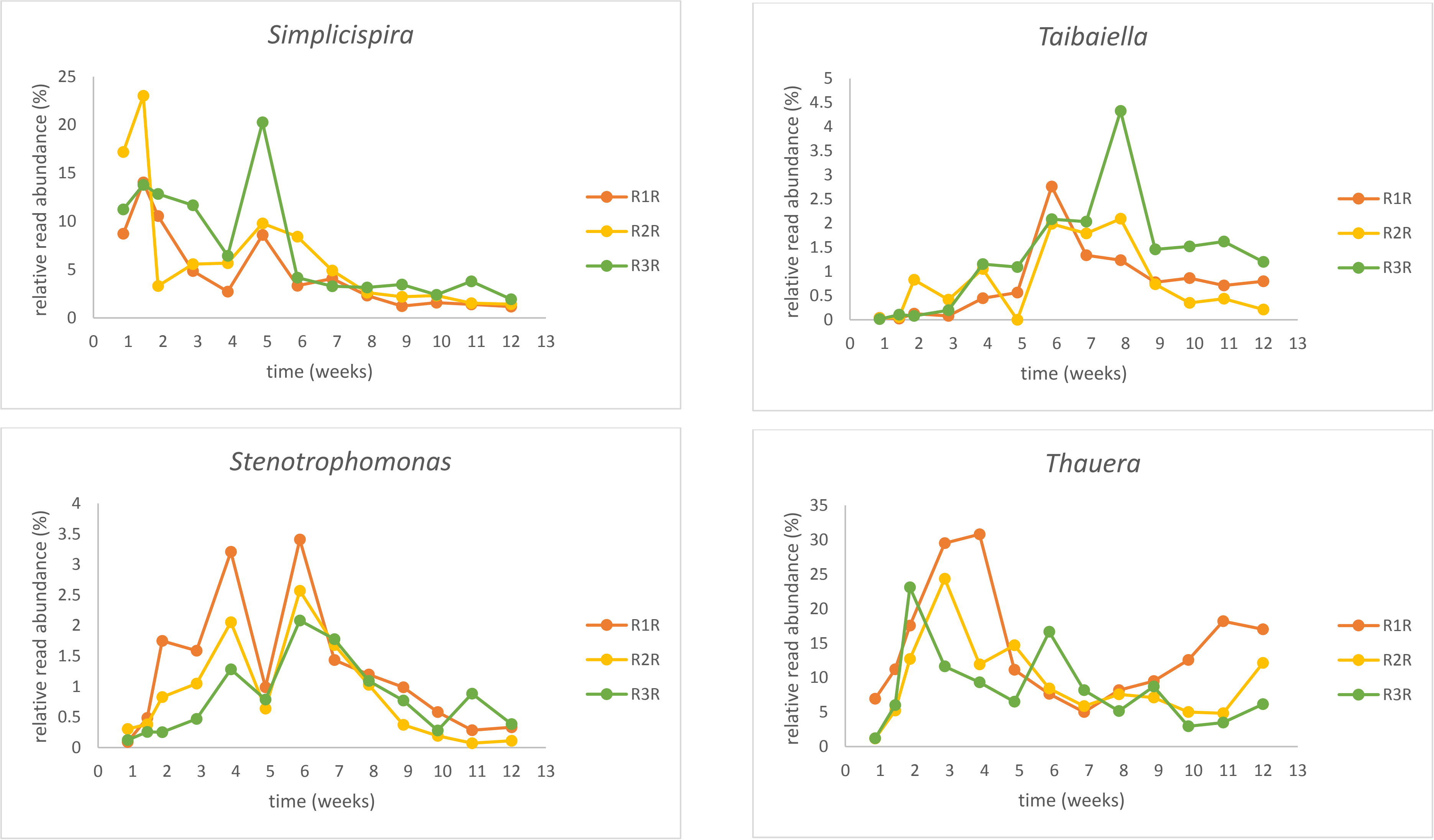

Supplementary Figure 5. - continued

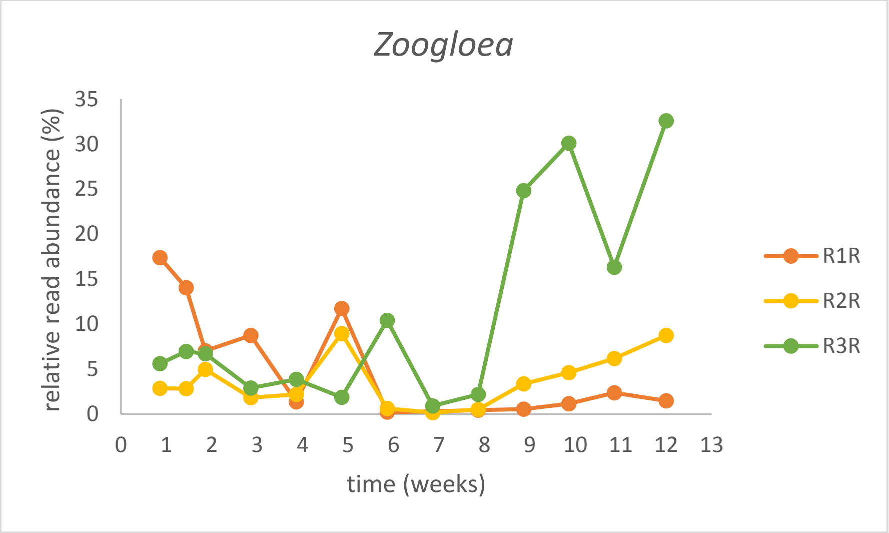

Supplementary Figure 6. Temporal variation in the relative read abundance of the nitrifying genera.

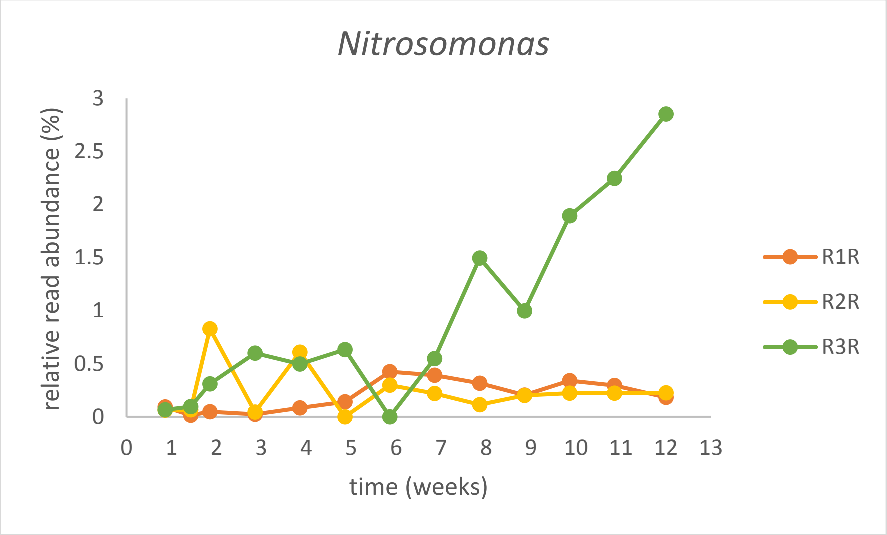

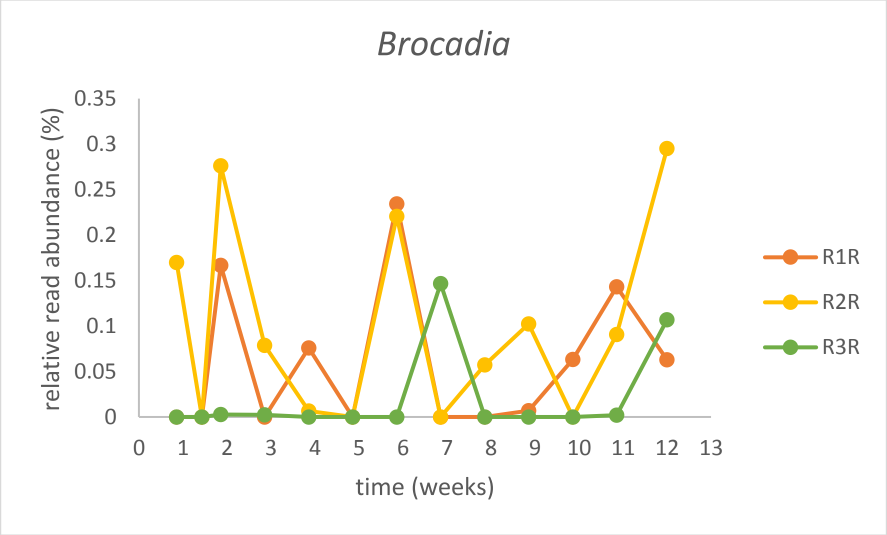

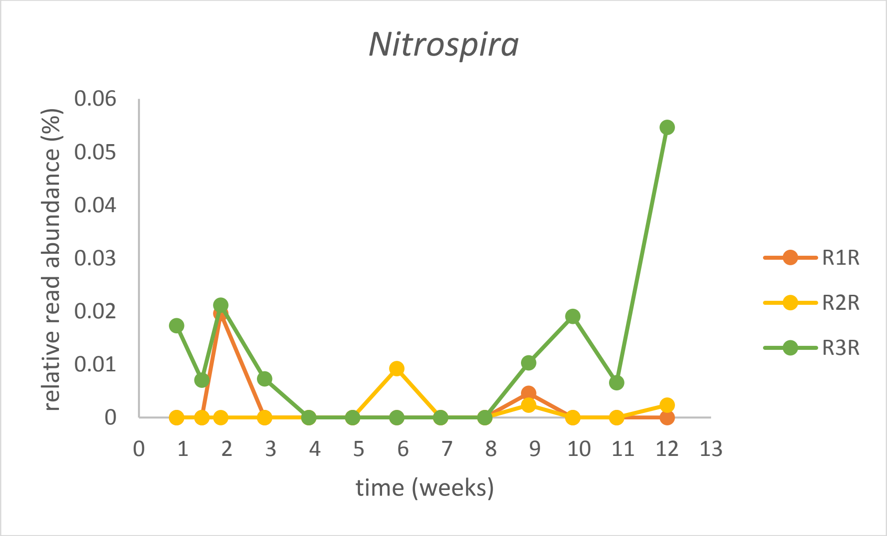

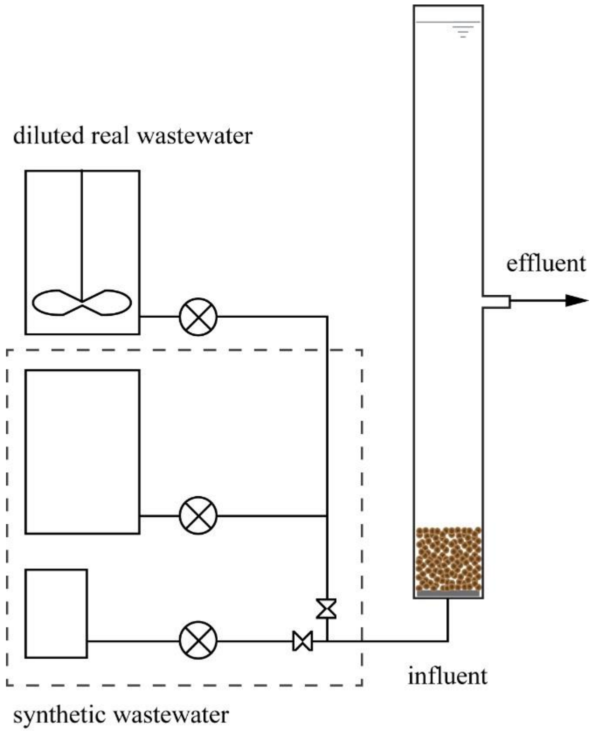

Supplementary Figure 7. Simplified scheme of the reactor design. The diluted real wastewater was stored in a cooled container (5°C), and mixed for 5 minutes before filling to ensure homogenous influent quality. The synthetic wastewater (dashed line) consisted of a concentrated acetate solution and a more dilute solution containing all other salts – this was necessary to avoid the depletion of acetate in the storage tanks.
